# IMU Networks for Trajectory Reconstruction in Logistics Applications

**DOI:** 10.3390/s23187838

**Published:** 2023-09-12

**Authors:** João Silva Sequeira

**Affiliations:** Institute for Systems and Robotics, Instituto Superior Técnico, Lisbon University, 1049-001 Lisbon, Portugal; joao.silva.sequeira@tecnico.ulisboa.pt

**Keywords:** trajectory reconstruction, Inertial Measurement Unit, Linear Matrix Inequalities, covariance intersection, target following

## Abstract

This paper discusses the use of networks of Inertial Measurement Units (IMUs) for the reconstruction of trajectories from sensor data. Logistics is a natural application domain to verify the quality of the handling of goods. This is a mass application and the economics of logistics impose that the IMUs to be used must be low-cost and use basic computational devices. The approach in this paper converts a strategy from the literature, used in the multi-target following problem, to reach a consensus in a network of IMUs. This paper presents results on how to achieve the consensus in trajectory reconstruction, along with covariance intersection data fusion of the information obtained by all the nodes in the network.

## 1. Introduction

Logistics is a booming service industry, including the flow of goods between origin and consumption points (see, for instance, [1]). With the diversity of goods being transported between any two points around the world, one of the quality indicators used by logistics operators is the way goods are manipulated during their transportation. Poor handling may result in damages and/or liability costs to insurance and/or logistics operators. Being able to analyze the movement of the goods upon delivery, i.e., having a traceability property, is recognized as strategic [2].

In engineering terms, this amounts to analyzing the trajectories the goods are subject to during transportation, i.e., the trajectories resulting from the way they are manipulated during transportation.

The trajectory reconstruction problem is well known in, for example, the aerospace industry. Using information from an IMU attached to the body of the spacecraft, its trajectory can be reconstructed using the velocity and acceleration data and assuming a good model of the spacecraft. Essentially, this amounts to an optimization problem, where the reconstructed trajectory must be such that the generated data is close to the observed data. The literature on this problem is vast, see, for instance, [3] on using multiple models and Unscented Kalman Filtering (UKF), [4] on using Extended Kalman Filtering (EKF) techniques and inertial measurements data, [5] on using image data, and [6] on using Gaussian regression techniques and GPS data.

The application to this logistics problem introduces additional constraints; namely, the sensing must be really cheap (i.e., the quality of the accelerometers and rate-gyros is nowhere close to that used in the aerospace industry), and the rigidity of the goods is only loosely verified. For example, goods such as food and domestic appliances are often transported in cardboard boxes and, hence, rigidity is valid under the assumption that the handling forces are relatively small or highly localized to avoid damages. Moreover, the placement of the sensors will, in general, be made by unskilled/carefree operators, which may lead to devices malfunctioning and, hence, redundancy, as with using multiple (similar) devices, is likely to increase the amount of valid information for trajectory reconstruction.

The vision of this paper is to implement this redundancy in that of a network of low-cost IMUs that can be glued and forgotten in typical packages (made out of cardboard, wood, plastic, metal, etc.) preserving, as much as possible, the rigidity property. These IMUs can either be placed: (i) forming a regular, pre-defined pattern, with the packages specifying precise areas of where to “glue” the devices, or (ii) distributed through the package arbitrarily. Placing the devices must not require any special expertise. The first scenario is possible in packages of standardized dimensions and limits the need for calibration procedures. In the second scenario, some calibration procedures may be necessary prior to the start of the transportation. For the sake of simplicity, this paper assumes that the whole IMUs in the network are placed at the same point. This does not imply any lack of generality; in a real scenario, with all the IMUs placed in different (and rigid) locations, the reconstructed trajectories would differ among them by constant homogeneous transformations.

Having multiple IMUs gathering data and combining it to reconstruct the trajectory may, in principle, improve the robustness of the solution due to the following reasons: (i) the amount of data increases, possibly containing richer information, and (ii) redundancy helps deal with sensor fails. Combining a posteriori the solutions found, from the data collected from each single IMU, e.g., using covariance intersection or some other averaging method, is a possibility and does not require the exchange of information among the nodes.

The alternative, followed in this paper, is to have a network of nodes, with each including: (i) sensory information from a local IMU, and (ii) communication with other distributed nodes (of a similar architecture) to exchange information and combine it locally to improve the reconstruction (this paper refers to a network of IMUs as these devices are the key component of the nodes). The main research question addressed is then whether or not using networks of IMUs improves trajectory reconstruction quality over a single IMU. A secondary research question is related to the framework defining the admissible protocols to exchange information among the IMU network.

The novel approach presented here follows from a multi-target tracking problem first presented in [7], re-interpreted as a consensus generation in a network of IMUs. The motivation to use this formulation comes from the proven optimality of the framework in [7] in the context of the multi-tracking problem.

This paper is organized as follows. Section 2 illustrates a single, simulated IMU, establishing a baseline performance for comparison with the consensus approach. Section 3 presents a quick view of the work in [7], which serves as a basis for this work, focusing on the consensus features and clarifying the re-interpretation of the key terms. Moreover, the feasibility problem, i.e., the existence of a strategy for the exchange of information, such that all the nodes in the network can provide estimates of the reconstruction, is discussed using the Linear Matrix Inequalities (LMI) framework. Section 4 illustrates the evolution of the performance indicators of the simulated nodes recovered from the network framework when the communications among them are inhibited. Section 5 presents simulation results with simple networks with two and three nodes. Section 6 discusses the improvement of the network consensus over the reconstruction by the independent nodes. Section 7 presents the results of the fusion of the trajectories reconstructed by each node using covariance intersection. Finally, Section 8 concludes this paper with a discussion on the feasibility of the approach and points to future research.

## 2. Baseline IMU

Given an arbitrary trajectory that serves as the ground truth, the corresponding linear and angular accelerations can be easily obtained if the inertia properties of the body (assumed rigid) moving over the trajectory are known. Thus, to accurately simulate the sensors to be glued to the logistics packages, knowledge of the inertia properties of the package is required beforehand.

In practical terms, full knowledge about the package inertia will seldom be available (though the mass and the physical dimensions can be easily known a priori as they are the parameters that directly influence the transportation cost). Therefore, while the observations reflect the real properties of the packages, the reconstruction of the trajectories has only a limited amount of that knowledge available. However, it should be emphasized that the estimation of the mass/inertia properties can also be completed using IMU data. This is a well-known problem in the aerospace industry, see, for instance, Ref. [8] on using recursive least squares, Ref. [9] on using continuous system identification techniques to identify the dynamics of the Hubble telescope, Ref. [10] on using the LMI framework and formulating a least squares problem, and Refs. [11,12] on using the least squares formulation combined with S-estimators for the identification of spacecraft parameters.

The basic approach to estimating the trajectory from the velocities and accelerations is to use simple integrator models. Given a trajectory expressed in terms of the position and orientation coordinates, x, y, z, α, β, γ, the corresponding signals from the accelerometers and rate-gyros can be obtained from basic expressions describing the geometry of the trajectory:

(1) $$\begin{array}{c} \ddot{x}=a_{x}\\ \ddot{y}=a_{y}\\ \ddot{z}=a_{z} \end{array}$$

for the translation and

(2) $$\dot{R}=\left[\begin{array}{ccc} 0 & -\omega_{z} & \omega_{y}\\ \omega_{z} & 0 & -\omega_{x}\\ -\omega_{y} & \omega_{x} & 0 \end{array}\right]$$

for the rotation, with *R*, the rotation matrix, expressing the orientation at each point in terms of Euler ZYX angles, which can be written as follows:

(3) $$\left[\begin{array}{c} \dot{\alpha }\\ \dot{\beta }\\ \dot{\gamma } \end{array}\right]={\left[\begin{array}{ccc} 0 & -s_{\alpha } & c_{\alpha }\;c_{\beta }\\ s_{\alpha }\;c_{\beta }\;\left(c_{\alpha }-c_{\alpha }\right) & c_{\alpha } & s_{\alpha }\;c_{\beta }\\ 1 & 0 & -s_{\beta } \end{array}\right]}^{-1}\;\left[\begin{array}{c} \omega_{x}\\ \omega_{y}\\ \omega_{z} \end{array}\right]$$

where $a_{x},\;a_{y},\;a_{z},\;\omega_{x},\;\omega_{y},\;\omega_{z}$ are the corresponding sensor outputs, i.e., the linear accelerations and angular velocities, respectively. Also, $s_{\theta },\;c_{\theta }$ stand for sin(θ) and cos(θ), respectively. The purpose of these expressions is to yield a simulation tool and, without the lack of generality, they implicitly assume a unit mass body. To improve realism, noise can be added to the outputs of these sensors.

### 2.1. Dead Reckoning Experiments

**Figure 1:**
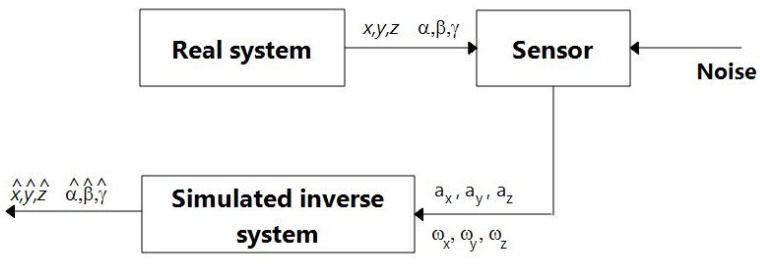
Dead reckoning trajectory reconstruction. Good estimates can be obtained if the model of the system is accurate and the effect of noise is minimized.

The purpose of these experiments is as follows: (i) to show the effects of noise when no compensation strategy is used, and (ii) to serve as the baseline to compare with the network experiments. Using sensor data obtained from (1) and (3), corresponding to some reference trajectory, and a dead reckoning model, the goal is to reconstruct the reference trajectory (this amounts to using an inverse model, as shown in Figure 1).

**Figure 2:**
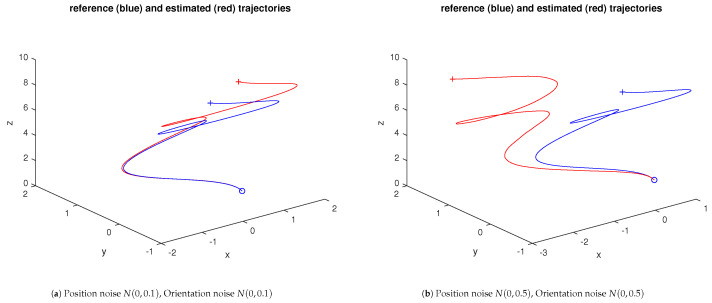
Dead reckoning test; x-y-z trajectory (orientation trajectory not shown). The ∘ and + marks stand for the start and end of the trajectories.

Sensor uncertainties make the reconstructed trajectory deviate from the real one. Figure 2 illustrates the effects of such situations for two levels of position noise.

From the perspective of a logistics application, the dead reckoning approach above may still be a valid option if the disturbances are limited and only specific events are relevant to measure the quality of the handling, e.g., if the existence of strong non-smooth/disturbances areas is the quality indicator. In such cases, even if the reconstructed trajectory in the Cartesian 3D space differs significantly from the real one, the presence of the areas of strong disturbances can still be recognized.

The existence of drifts in the observed variables is another important disturbance factor. The poor assembly of a sensor and electronics issues are common causes of drifts. In the accelerometer, drift means that the dynamics of the acceleration are constantly integrating some value that is not being observed by the accelerometer. In the case of the rate-gyro, this means that the dynamics of the rotational velocity are constantly integrating some value that is not being directly observed by the gyro.

**Figure 3:**
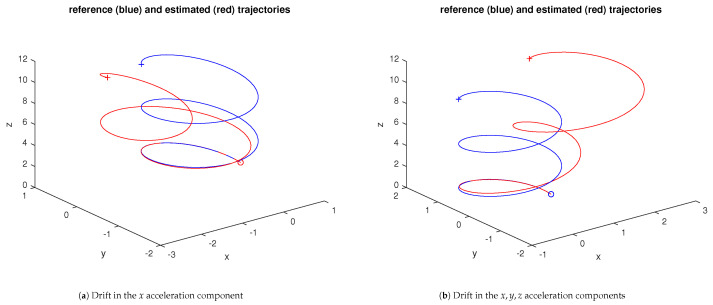
Dead reckoning experiment; spiral x-y-z trajectory under drift conditions (Wiener process). The ∘ and + marks stand for the start and end of the trajectories.

Figure 3 illustrates two experiments of the dead reckoning approach under constant drift conditions. The disturbances considered only affect the linear acceleration. In the first sample, they are given as follows:

$$a_{offset_{k}}=a_{offset_{k-1}}+\left[0.05*\mathrm{randn},0,0\right]$$

where “randn” stands for the usual function returning a random value with an N(0,1) distribution. In the second sample, all the coordinates are similarly affected by 0.05∗randn.

This is an example where the estimated trajectory is too different from the original one. However, depending on the specific requirements of the logistics application, the drift disturbances may not be an issue, e.g., as mentioned above if the purpose is the detection of harsh handling. Clearly, drifts are highly relevant if accurate trajectory reconstruction is required, which is the focus of the remainder of this paper.

### 2.2. Single IMU

**Figure 4:**
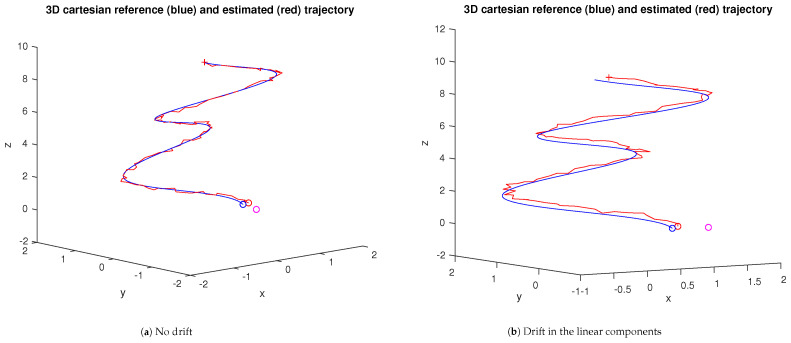
Single IMU sample for a biased spiral reference trajectory (position trajectory only), with/without drift disturbance. The blue and red ∘ and + marks stand for the start and end of the trajectories, respectively. The magenta ∘ stands for the initial unfiltered estimate.

In general, an IMU will include a filtering stage, often formed by an Extended Kalman Filter. The process and observation models can be easily obtained from (1) and (3). Figure 4 shows a sample of the reconstruction for a biased spiral reference trajectory, with the position and orientation N(0,0.1) process noises and N(0,0.01) and N(0,0.001) observation noises for the position and orientation, respectively.

From the results in Figure 4, a single IMU can already provide quality information about the real trajectory, even in the presence of mild disturbances. Similar to the dead reckoning option, the basic IMU in this example can already be useful in the logistics domain. However, the research goal for this paper is aimed at expanding the performance of the single IMU.

## 3. Background

This paper adopts the formulation from [7], developed in the context of a multi-target tracking problem, with the targets and measurements assumed to have a one-to-one relation. The notation follows the usual form of the continuous-discrete (CD) extended Kalman framework (see, for instance, [13] for a critical comment and the implementation issues of this form), with the prediction stage for the *i*th IMU node given by the following (see also [14] for a face-off between five different formulations):

(4) $$\begin{array}{c} x_{k|k-1}^{i}=f_{k-1}^{i}\left(x_{k-1|k-1}^{i}\right)\\ z_{k}^{i}=h_{k}^{i}\left(x_{k|k-1}^{i}\right)\\ P_{k|k-1}^{i}=F_{k-1}^{i}\;P_{k-1|k-1}^{i}{\left(F_{k-1}^{i}\right)}^{T}+Q_{k-1}^{i}, \end{array}$$

where $x^{i}$ stands for the state variables, $z^{i}$ the observations, $P^{i}$ the covariance matrix, $Q^{i}$ the observation noise covariance, *F* the Jacobian of *f*, and the modified update stage is as follows:

(5) $$\begin{array}{c} S_{k}^{i}=H_{k}^{i}\;P_{k|k-1}^{i}\;{\left(H_{k}^{i}\right)}^{T}+R_{k}^{i} \end{array}$$

(6) $$\begin{array}{c} K_{k}^{i}=P_{k|k-1}^{i}\;{\left(H_{k}^{i}\right)}^{T}\;{\left(S_{k}^{i}\right)}^{-1} \end{array}$$

(7) $$\begin{array}{c} x_{k|k}^{i}=x_{k|k-1}^{i}+K_{k}^{i}\;{\tilde{z}}_{k}^{i} \end{array}$$

(8) $$\begin{array}{c} P_{k|k}^{i}=P_{k|k-1}^{i}-K_{k}^{i}\;\left(1-\beta_{0}^{i}\right)\;S_{k}^{i}\;{\left(K_{k}^{i}\right)}^{T}+K_{k}^{i}\;{\bar{P}}_{k}^{i}\;{\left(K_{k}^{i}\right)}^{T} \end{array}$$

where *H* is the Jacobian of *h*, $R^{i}$ is the process noise covariance matrix, and the networking and consensus are expressed through the measurement uncertainties:

(9) $${\bar{P}}_{k}^{i}=\sum_{j=1}^{M_{k}}\beta_{j}^{i}\;{\tilde{z}}_{j,k}^{i}\;{\left({\tilde{z}}_{j,k}^{i}\right)}^{T}-{\tilde{z}}_{k}^{i}{\left({\tilde{z}}_{k}^{i}\right)}^{T},$$

using the $M_{k}$ measurements, ${\tilde{z}}_{j,k}^{i}$ (received by the *i*th IMU from the *j*th IMU, i.e., the *i*th IMU is connected to $M_{k}$, which are the other IMUs), and with the weighted innovations, is as follows:

(10) $$\begin{array}{c} {\tilde{z}}_{k}^{i}=\sum_{j=1}^{M_{k}}\beta_{j}^{i}\left(z_{j,k}^{i}-{\hat{z}}_{k}^{i}\right)\\ {\tilde{z}}_{j,k}^{i}=z_{k}^{i}-{\hat{z}}_{j,k}^{i}, \end{array}$$

where $\beta_{j}^{i}$ is the weight expressing the relevance of the measurement from node *j* to the estimate of the measurement by node *i*, $\beta_{0}^{i}$ is the weight expressing the relevance of the poor measurements ($\beta_{0}^{i}\to 1$ amounts to reducing the norm of $P_{k|k}^{i}$, forcing the covariance to decrease even if poor measurements are being fed into the system), ${\hat{z}}_{k}^{i}$ is the estimate of the measurement of the *i*th node at instant *k*, that is, ${\hat{z}}_{k}^{i}=h_{k}^{i}\left(x_{k|k-1}^{i}\right)$, and $z_{j,k}^{i}$ is the actual measurement of node *j* at instant *k* reaching the *i*th IMU.

The topology of the network is assumed fixed. In [7], the $\beta_{j}^{i}$ parameters were interpreted in terms of a marginal probability of association between node *j* and target *i*. The $\beta_{0}^{i}$ is used to quantify the misdetection of a target (1/0 for misdetected/detected). However, for the purpose of this paper, this interpretation is not useful as the association between the nodes and measurements can be known a priori. Instead, these parameters can be seen as follows: (i) the communications protocol (as in consensus problems), and (ii) scaling weights for the measurements. Also, as in the original problem, the number of measurements, $M_{k}$, can vary along time (or measurement scans) *k*.

The formulation from [7] is, essentially, an EKF with a modified update of the covariance matrix (through the $\beta_{0}$ factor that scales the innovation residual covariance in (5), and a weighted combination of the innovations from the whole network weighted by the $\beta_{j}^{i}$. Also, (8) dumps the term explicit in the noise $K_{k}^{i}\;R_{k}^{i}\;{\left(K_{k}^{i}\right)}^{T}$, and the symmetric term ${\left(K_{k}^{i}H_{k}^{i}\right)}^{T}+K_{k}^{i}H_{k}^{i}$ affecting $P_{k|k-1}^{i}$ in the CD formulation of the EKF.

This innovates relative to the CD-EFK procedure in that the covariance of each unit now has feedback from the innovations produced by the network. Moreover, the tuning “knob” $R_{k}^{i}$ in the CD-EKF formulation is now replaced with the $\beta_{j}^{i}$ and $\beta_{0}^{i}$ parameters. These can also be seen as the mediator/protocol/scaling factors between the nodes.

For the sake of readability, the expression for the covariance update in the CD-EKF formulation, and a re-writing of (8) to highlight the differences to the CD-EKF covariance update equation, is presented below.

(11) $$\begin{array}{c} P_{k|k}^{i}=\left(I-K_{k}^{i}\;H_{k}^{i}\right)\;P_{k|k-1}^{i}\;{\left(I-K_{k}^{i}\;H_{k}^{i}\right)}^{T}+K_{k}^{i}\;R_{k}^{i}\;{\left(K_{k}^{i}\right)}^{T}\;\;\;\;\;\;\;\;\;\;\;\;\;\;\;\;\;\;\;\;\;\;\\ =P_{k|k-1}^{i}+K_{k}^{i}\;S_{k}^{i}{\left(K_{k}^{i}\right)}^{T}-K_{k}^{i}\;H_{k}^{i}\;P_{k|k-1}^{i}-P_{k|k-1}^{i}\;{\left(K_{k}^{i}\;H_{k}^{i}\right)}^{T} \end{array}$$

The renewed one, where ${\tilde{z}}_{j,k}$ and ${\tilde{z}}_{j,k}^{i}$ are $N\times M_{k}$ matrices is as follows:

(12) $$\begin{array}{c} P_{k|k}^{i}=P_{k|k-1}^{i}-K_{k}^{i}\;S_{k}^{i}\;{\left(K_{k}^{i}\right)}^{T}+\;\;\;\;\;\;\;\;\;\;\;\;\;\;\;\;\;\;\;\;\\ \;\;\;\;+K_{k}^{i}\;\left(\sum_{j=1}^{M_{k}}\beta_{j}^{i}\;{\tilde{z}}_{j,k}^{i}\;{\left({\tilde{z}}_{j,k}^{i}\right)}^{T}\right){\left(K_{k}^{i}\right)}^{T}-K_{k}^{i}\;{\tilde{z}}_{k}^{i}{\left({\tilde{z}}_{k}^{i}\right)}^{T}\;{\left(K_{k}^{i}\right)}^{T}+\beta_{0}^{i}\;K_{k}^{i}\;S_{k}^{i}\;{\left(K_{k}^{i}\right)}^{T} \end{array}$$

The first two terms on the righthand side of (12) are also present in (11). The third and fourth terms account for the network exchanges and are the additional “tuning knob”. The fifth term dampens the second term and provides additional control.

The convergence of (12) can be achieved by the careful selection of $\beta_{0}^{i}$ and $\beta_{j}^{i}$, namely ensuring that $P_{k|k}^{i}$ is a contracting sequence converging to some fixed point (see [15]). This means:

$$\exists k_{0}>0,\;\alpha >0,\;\gamma >0\;:\;\forall k>k_{0},\;\;∥P_{k|k}^{i}-P_{k|k-1}^{i}∥\leq \alpha \;e^{-\gamma \;(k-k_{0})},$$

or, alternatively,

(13) $$\begin{array}{c} \exists k_{0}>0,\;\alpha >0,\;\gamma >0\;:\;\forall k>k_{0},\;\;\\ ∥-\left(1-\beta_{0}^{i}\right)K_{k}^{i}\;S_{k}^{i}\;{\left(K_{k}^{i}\right)}^{T}+\sum_{j=1}^{M_{k}}\beta_{j}^{i}\;\left(K_{k}^{i}\;{\tilde{z}}_{j,k}^{i}\;{\left({\tilde{z}}_{j,k}^{i}\right)}^{T}\;{\left(K_{k}^{i}\right)}^{T}\right)-K_{k}^{i}\;{\tilde{z}}_{k}^{i}{\left({\tilde{z}}_{k}^{i}\right)}^{T}\;{\left(K_{k}^{i}\right)}^{T}∥\leq \alpha \;e^{-\gamma \;(k-k_{0})}, \end{array}$$

or, in a more compact form to highlight the terms involved in the consensus, with $A_{k}^{i}=\left(1-\beta_{0}^{i}\right)K_{k}^{i}\;S_{k}^{i}\;{\left(K_{k}^{i}\right)}^{T}$, $C_{k}^{i}=K_{k}^{i}\;{\tilde{z}}_{k}^{i}{\left({\tilde{z}}_{k}^{i}\right)}^{T}\;{\left(K_{k}^{i}\right)}^{T}$, and $F_{j,k}^{i}=K_{k}^{i}\;{\tilde{z}}_{j,k}^{i}\;{\left({\tilde{z}}_{j,k}^{i}\right)}^{T}{\left(K_{k}^{i}\right)}^{T}$,

(14) $$\begin{array}{c} \exists k_{0}>0,\;\alpha >0,\;\gamma >0\;:\;\forall k>k_{0},\;\;\;\;\;\;\;\;\;\;\;\;\;\;\;\;\;\;\;\;\;\;\;\;\;\;\\ ∥-A_{k}^{i}-C_{k}^{i}+\sum_{j=1}^{M_{k}}\beta_{j}^{i}\;F_{j,k}^{i}∥<\alpha \;e^{-\gamma \;(k-k_{0})}. \end{array}$$

At each time step, a new solution must be produced so that the convergence of the covariance matrix can be controlled. This means: (i) the consensus will be adjusting over time, as the $\beta_{j}^{i}$ will (in general) be changing, and (ii) the tradeoff values for α,γ (that can be constant over time) have to be selected. However, one must ensure that the α,γ can be made unique during the whole duration of the trajectory, despite the changes in the $\beta_{j}^{i}$.

This makes:

(15) $$Z_{k}^{i}=\frac{1}{\alpha }e^{\gamma (k-k_{0})}\left(-A_{k}^{i}-C_{k}^{i}+\sum_{j=1}^{M_{k}}\beta_{j}^{i}\;F_{j,k}^{i}\right),$$

the solution of

(16) $$∥Z_{k}^{i}∥<1,$$

which can be obtained using the LMI form (see, for instance, [16], pp. 7–8, on computing maximum singular values) as

(17) $$\left[\begin{array}{cc} I & Z_{k}^{i}\\ {\left(Z_{k}^{i}\right)}^{T} & I \end{array}\right]>0.$$

The complete solution for the convergence of (12) requires an additional LMI that imposes the positive definiteness of $P_{k|k}^{i}$ given that $P_{k|k-1}^{i}$ is assumed positive definite is as follows:

(18) $$P_{k|k-1}^{i}-A_{k}^{i}-C_{k}^{i}+\sum_{j=1}^{M_{k}}\beta_{j}^{i}\;F_{j,k}^{i}≻0,$$

or, more compactly, with $D_{k}^{i}=P_{k|k-1}^{i}-A_{k}^{i}-C_{k}^{i}$,

(19) $$D_{k}^{i}+\sum_{j=1}^{M_{k}}\beta_{j}^{i}\;F_{j,k}^{i}≻0.$$

Therefore, if (17) and (19), with the $\beta^{i}$ variables, are both feasible at each time step, *k*, for some choice of $\beta_{j}^{i}$, then there is a consensus solution to the network from the point of view of the *i*th node, which ensures the convergence of $P_{k|k}^{i}$. A solution for the full network requires the feasibility of the above LMI problems for all the nodes.

The LMI feasibility can be tested by multiple algorithms from general ones, Refs. [17,18] on ellipsoid algorithms, to specific ones, [19] for large and sparse LMIs or Ref. [20] on stochastic algorithms. Several software packages to solve LMIs are available, i.e., the Scilab-based LMITOOL [21], the Matlab-based YALMIP [22], and the Matlab LMI toolbox.

These LMI problems are non-convex (the presence of the affine term in (19) is enough to make it non-convex) and, hence, finding optimal parameters using standard LMI solvers is, in general, an issue. Relaxation techniques are commonly used to solve non-convex problems and obtain either good solutions or pseudo-solutions from which the approximations to the good ones can be derived (see, for instance, [23,24,25]). The approach proposed here uses a basic form of relaxation, consisting in scaling some terms until a solution can be found.

## 4. Simulation Experiments

Making $\beta_{0}^{i}=0$ and $\beta_{j}^{i}=1,\;\mathrm{if}\;i=j,\;\mathrm{and}\;0,\mathrm{otherwise}$, then ${\tilde{z}}_{k}^{i}={\tilde{z}}_{j,k}^{i}$ for j=i, recovering a set of isolated nodes:

(20) $$\begin{array}{c} E_{k}^{i}\equiv P_{k|k-1}^{i}-K_{k}^{i}\;S_{k}^{i}\;{\left(K_{k}^{i}\right)}^{T}>0 \end{array}$$

and the convergence condition becomes:

(21) $$\begin{array}{c} ∥\frac{1}{\alpha }e^{\gamma (k-k_{0})}\left(-K_{k}^{i}\;S_{k}^{i}\;{\left(K_{k}^{i}\right)}^{T}\right)∥<1. \end{array}$$

Given that all the matrices in (20) are norm-bounded, then the second condition can always be verified for a large enough α and a small enough γ.

The noise covariance $R_{k}^{i}$ bounds the definiteness of (20) (in the best possible scenario $R_{k}^{i}=0$; in general, this is not a free parameter as it must be chosen in connection with the sensors, though it can also act as a tuning knob to some extent). The matrix $S_{k}^{i}$ is, by construction, always positive definite and, hence, the quadratic term $K_{k}^{i}\;S_{k}^{i}\;{\left(K_{k}^{i}\right)}^{T}$ is also positive definite. By introducing a scale factor $\xi_{k}^{i}\in \left[-1,1\right]$ in $K_{k}^{i}$, the LMI (21) dominates that in (20), i.e., the set of solutions $P_{k|k-1}^{i}$ of (21) contains those of (20) (see [26] for detailed analysis and the results related to LMI dominance).

Given the following scaled expression:

(22) $$P_{k|k-1}^{i}-{\left(\xi_{k}^{i}\right)}^{2}\;K_{k}^{i}\;S_{k}^{i}\;{\left(K_{k}^{i}\right)}^{T}>0,$$

as $\xi_{k}^{i}\to \pm 1$, the solution $P_{k|k-1}^{i}$ converges to that of the unscaled expression. Also, $\xi_{k}^{i}\to 0$, $P_{k|k}^{i}\to P_{k|k-1}^{i}$, which is construction positive definite.

Given the low complexity of the scaled expression, its feasibility relative to the $\xi_{k}^{i}$ parameter can be obtained from the eigenvalues/eigenvectors of the matrix:

(23) $$G_{k}^{i}\equiv P_{k|k-1}^{i}\;{\left(\xi_{k}^{i}\right)}^{-2}{\left(K_{k}^{i}\;S_{k}^{i}\;{\left(K_{k}^{i}\right)}^{T}\right)}^{-1}.$$

The positiveness of all the eigenvalues means $G_{k}^{i}>0$. As the $\xi_{k}^{i}$ varies, the eigenvalues of the relaxed matrix are linearly related to those of $G_{k}^{i}$.

**Figure 5:**
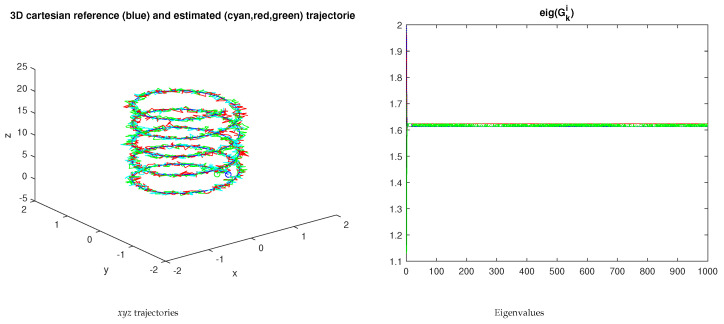
Trajectories and eigenvalues evolution for 3 independent nodes. The positive definiteness of the $G_{k}^{i}$ matrix is clear. The ∘ and + marks stand for the start and end of the trajectories, respectively.

Figure 5 shows an example of the evolution of the eigenvalues using $\xi_{k}^{i}=1$.

As expected, the quality of this solution, using the EKF, is clearly superior to that in the example of dead reckoning.

Whenever the nodes are not independent and the network must reach a consensus, the feasibility must be checked directly using (17) and (19).

## 5. Network Consensus Experiments

**Figure 6:**
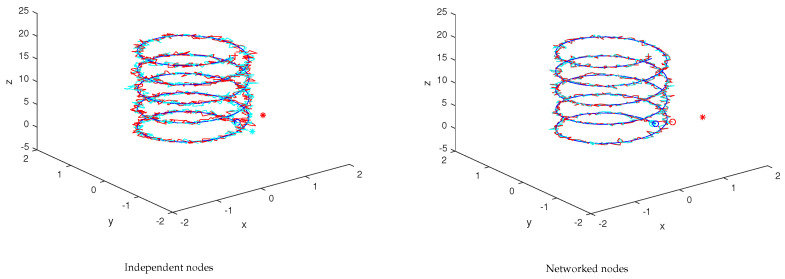
Samples of the two-node network (xyz trajectories). The ∘ and + marks stand for the trajectory starting and final points, respectively, with the ∗ mark the initial unfiltered estimate.

Figure 6 shows the 3D space trajectories for the two nodes operating independently and as a network. All the IMUs are assumed to be time-synchronized, i.e., the simulation loop time is the same for all of them. For the independent nodes test, $\beta_{0}^{i}=0,\;i=1,2$, $\beta_{1}^{1}=1,\;\beta_{2}^{1}=0,\;\beta_{1}^{2}=0,\;\beta_{2}^{2}=1$. For the networked nodes test, $\beta_{0}^{i}=0.01,\;i=1,2$, $\beta_{1}^{1}=0.6,\;\beta_{2}^{1}=0.4,\;\beta_{1}^{2}=0.4,\;\beta_{2}^{2}=0.6$. Any of the IMU trajectories can be used for reconstruction purposes.

**Figure 7:**
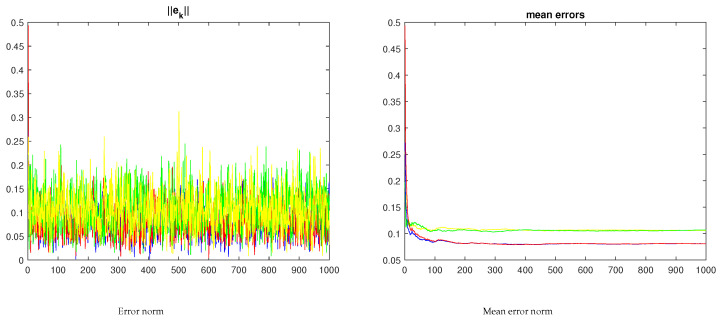
Two-node error evolution for the samples in Figure 6 (the red/blue curves refer to the networked nodes; the yellow/green curves in the righthand plot refer to the independent nodes under no-consensus).

Figure 7 shows the evolution of the two-norm of the xyz trajectory and of the respective mean. Clearly, the mean error when the nodes operate cooperatively is lower than when operating independently.

**Figure 8:**
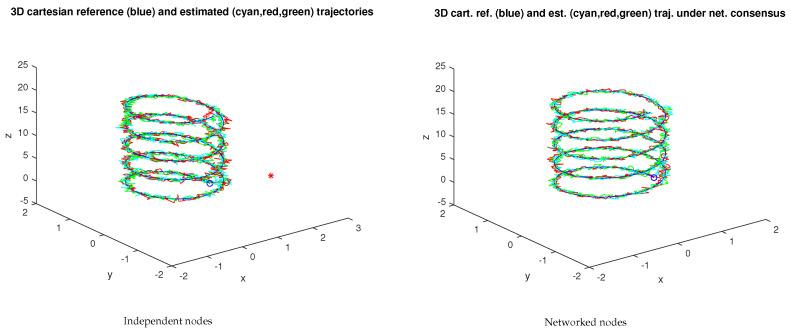
Samples of the three-node network (xyz trajectories). The ∘ and + marks stand for the trajectory starting and final points, respectively, with the ∗ mark the initial unfiltered estimate.

**Figure 9:**
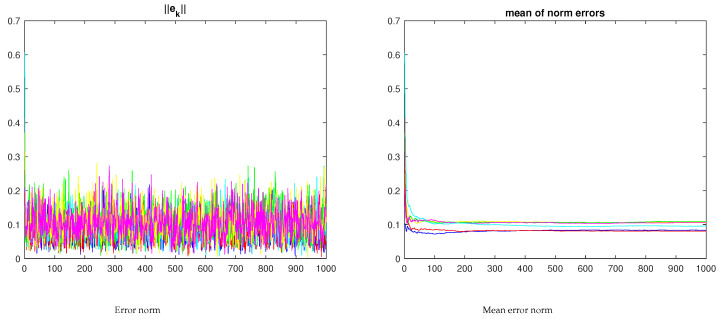
Three-node network error evolution for the sample in Figure 8 (the red/blue/cyan curves refer to the networked nodes; the yellow/green/magenta curves in the righthand plot refer to the independent nodes under no-consensus).

Figure 8 and Figure 9 show a similar experiment with three nodes. For the independent nodes test, $\beta_{0}^{i}=0,\;i=1,2,3$, $\beta_{1}^{1}=1,\;\beta_{1}^{2}=0,\;\beta_{2}^{1}=0,\;\beta_{2}^{2}=1,\;\beta_{2}^{3}=0,\;\beta_{3}^{1}=0,\;\beta_{3}^{2}=0,\;\beta_{3}^{3}=1$. For the networked nodes test, $\beta_{0}^{i}=0.01,\;i=1,2,3$, $\beta_{1}^{1}=0.7,\;\beta_{2}^{1}=0.01,\;\beta_{3}^{1}=0.01$, $\beta_{1}^{2}=0.01,\;\beta_{2}^{2}=0.7,\;\beta_{3}^{2}=0.01,\;\beta_{1}^{3}=0.01,\;\beta_{2}^{3}=0.01,\;\beta_{3}^{3}=0.8$. The protocol for the network experiments, i.e., the set of $\beta^{i}$ gains, was chosen to have each IMU privileging its own estimates but still accounting for the other nodes.

For the three-node network, the mean error of the xyz trajectory of the networked nodes is also lower than the error for the independent nodes. However, the increase in the number of IMUs does not immediately result in an increase in performance, i.e., a lower mean error norm.

**Figure 10:**
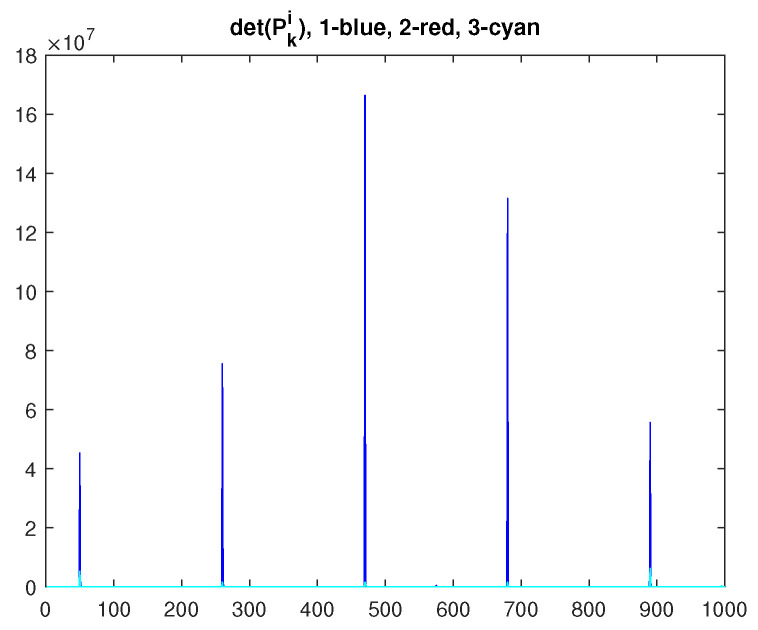
Feasibility evolution ($\alpha =1000,\;\gamma =10^{-5}$) for the 3-node experiment.

The evolution of the feasibility conditions, i.e., (17) and (19), is shown in Figure 10.

**Figure 11:**
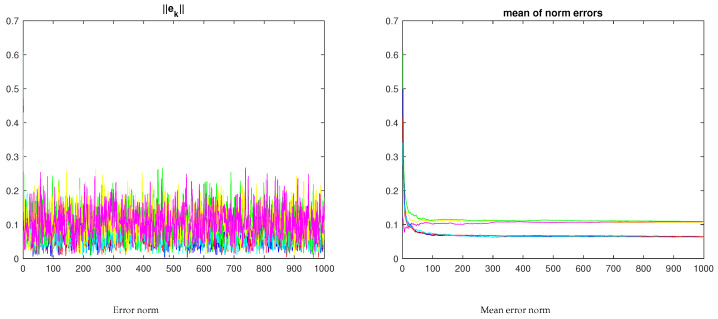
Three-node network error evolution for an alternative $\beta^{i}$ protocol (the red/blue/cyan curves refer to the networked nodes; the yellow/green/magenta curves in the righthand plot refer to the independent nodes under no-consensus).

Alternative protocol values, e.g., increasing the contributions of the surrounding nodes with $\beta_{0}^{i}=0.01,\;i=1,2,3$, $\beta_{1}^{1}=0.5,\;\beta_{2}^{1}=0.2,\;\beta_{3}^{1}=0.1,\;\beta_{1}^{2}=0.2,\;\beta_{2}^{2}=0.5,\;\beta_{3}^{2}=0.2$, $\beta_{1}^{3}=0.1,\;\beta_{2}^{3}=0.2,\;\beta_{3}^{3}=0.5$, continues to yield a lower error for the consensus version (see Figure 11). These values were defined empirically, and they ensure LMI feasibility. Given that the trajectory reconstruction will, in general, be made offline, finding alternative admissible protocols is not a problem. The alternative workflow to finding a solution to the LMI feasibility problem is to use an empirical approach, starting with independent nodes and gradually increasing the contribution of each node to the estimates of the neighbors, checking the LMI feasibility at each step.

Figure 9b and Figure 11b illustrate the dependence of the mean error norm from the $\beta^{i}$ values. Moreover, it provides the ground for an optimization problem on the space of the feasible $\beta^{i}$. It is worth noting that increasing the exchange of information, i.e., increasing the $\beta^{i}$, had the effect of improving the performance.

From a practical perspective, if, during an online reconstruction, the LMI problem is unfeasible, then simply use one of the independent units as a solution.

## 6. Networking for Improvement over Independent Nodes

As seen before, the feasibility of a network can be tied to an LMI. Also, the experiments in the previous section empirically show that networks have an advantage over independent nodes. The purpose of this section is to demonstrate this advantage, i.e., the difference between the tracking errors obtained in the networked and independent versions:

(24) $$\begin{array}{c} e_{k}^{i}=∥e_{k}^{i^{net}}∥-∥e_{k}^{i^{ind}}∥=∥r-x_{k|k}^{i^{net}}∥-∥r-x_{k|k}^{i^{ind}}∥\\ \;\;\;\;\;\;\;\;\;\;=∥r-x_{k|k-1}^{i^{net}}+K_{k}^{i^{net}}{\tilde{z}}_{k}^{i^{net}}∥-∥r-x_{k|k-1}^{i^{ind}}+K_{k}^{i^{ind}}{\tilde{z}}_{k}^{i^{ind}}∥, \end{array}$$

where *r* is the reference trajectory, $x_{k|k}^{i^{net}}$ is the trajectory reconstructed by a network, and $x_{k|k}^{i^{ind}}$ is a trajectory reconstructed by one node operating independently, verifying the averaging relation:

(25) $$\exists N:\;n>N,\;\frac{1}{n}\sum_{k=1}^{n}e_{k}^{i}<0.$$

For a discrete, time-varying, linear system, Lemma 3.1 in [27] states that $P_{k|k}^{i^{ind}}$ is bounded above and below, i.e., $\exists \mu_{l},\mu_{u}>0\;:\;\mu_{l}I<P_{k|k}^{i^{ind}}<\mu_{u}I$. $S_{k}^{i^{ind}}$ is a linear operator on $P^{i^{ind}}$ and, hence, it is also bounded above and below. $K_{k}^{i^{ind}}$ is also bounded as it depends on the bounded $P^{i^{ind}}$ and on ${\left(S_{k}^{i^{ind}}\right)}^{-1}$, which is also upper and lower bounded. Therefore, $x_{k}^{i^{ind}}$ is bounded and, also, the whole rightmost term in (24) can be written as follows:

$$L_{i}\;I<r-x_{k|k-1}^{i^{ind}}+K_{k}^{i^{ind}}{\tilde{z}}_{k}^{i^{ind}}<L_{s}\;I,$$

for some $L_{i},L_{s}>0$, and I is a suitable identity matrix.

Applying a two-norm on both sides of an inequality preserves the inequality, as this norm is monotone [28], and the righthand term can thus be assumed to be a bounded function:

(26) $$L_{i}<∥r-x_{k|k-1}^{i^{ind}}+K_{k}^{i^{ind}}{\tilde{z}}_{k}^{i^{ind}}∥<L_{s}.$$

The leftmost term in (24) is a function of the $\beta_{j}^{i}$ parameters. Without losing generality, one can represent this term as follows:

(27) $$∥r-x_{k|k-1}^{i^{net}}+K_{k}^{i^{net}}{\tilde{z}}_{k}^{i^{net}}∥\equiv ∥r-g(\beta )+\beta f(\beta )∥,$$

with f(·),g(·) as the adequate functions. Therefore, (24) can be written as follows:

(28) $$∥r-g(\beta )+\beta f(\beta )∥-L_{s}<e_{k}^{i}<∥r-g(\beta )+\beta f(\beta )∥-L_{i}.$$

Under the LMI feasibility conditions, f(·) and g(β) are bounded and, hence, by selecting small enough β constants, the rightand side of (28) approaches:

(29) $$∥r-g(\beta )∥-L_{i}.$$

The feasibility of the LMI problem ensures the asymptotic convergence of $g\left(\beta \right)\equiv x_{k|k-1}^{i}\to x_{k|k}^{i}$ and, hence, it is clear that, if the networked version converges to the reference trajectory *r*, the $e_{k}^{i}$ are negative.

**Figure 12:**
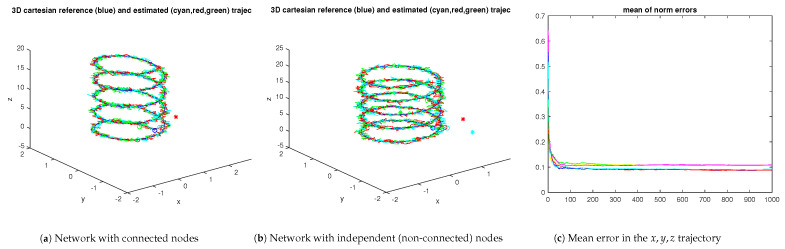
A 3-node network with all nodes subject to unmodeled drift (the red/blue/cyan curves refer to the networked nodes; the yellow/green/magenta curves in the righthand plot refer to the independent nodes under no-consensus). The ∘ and + marks stand for the initial and final positions, respectively. The ∗ mark indicates the initial unfiltered estimates.

The consensus approach can also be beneficial in the case of structured unmodeled disturbances, as in the case of unmodeled drift in the sensors. From Figure 12, the consensus approach retains the good property of low mean error when compared with the non-consensus approach.

**Figure 13:**
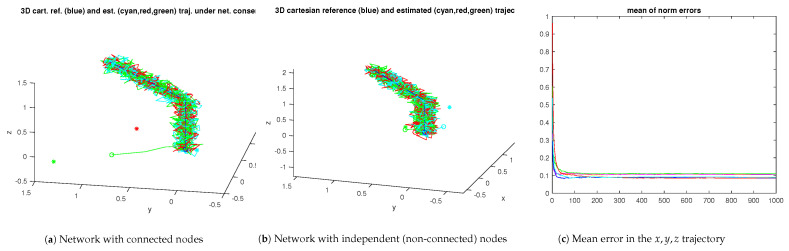
A 3-node network with all nodes subject to unmodeled drift reconstructing a piecewise linear reference trajectory (the red/blue/cyan curves refer to the networked nodes; the yellow/green/magenta curves in the righthand plot refer to the independent nodes under no-consensus). The circ and +marks stand for the start enad end positions, respectively. The ∗ mark stands for the initial unfiltered estimates.

Figure 13 illustrates the performance in the case of a piecewise linear (two trunks) reference trajectory. As before, the mean error property is verified.

## 7. Network Fusion Using Covariance Intersection

The framework above leads to each IMU producing its own estimate, each of which is better than the independent estimates. A single estimate can be obtained by applying the covariance intersection procedure [29] (the inverse covariance matrices are combined using a linear convex law, with a fixed parameter ω). For the two-node case, the procedure can be applied directly; a parameter $\omega_{1}$ controls the relative importance between the two data sources being fused. For the three-node case, the expressions used for the two-node case can be cascaded, i.e., a first stage fuses two nodes and a second stage fuses the result with the third node. A $\omega_{2}$ parameter controls the importance between nodes one and two, and node three. In this case, $\omega_{1,2}=0.5$, meaning that the data sources in each stage have the same relative importance.

**Figure 14:**
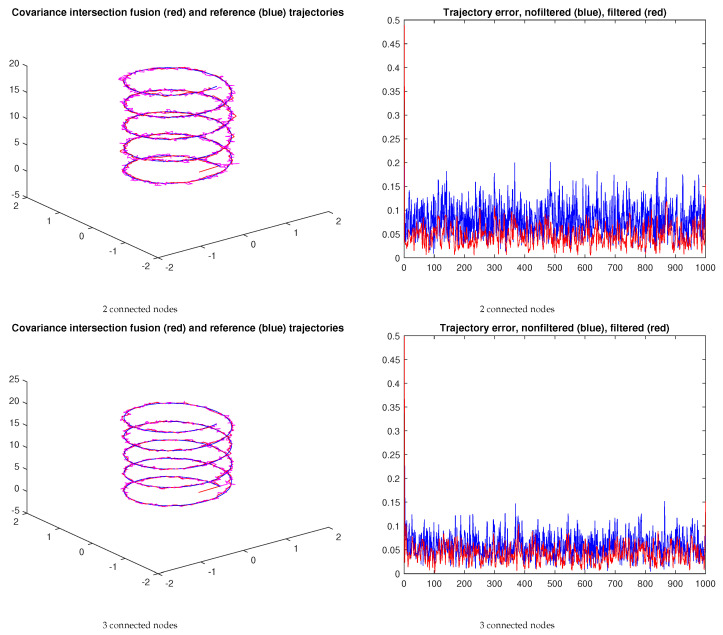
Trajectories and tracking errors resulting from fusion using covariance intersection ($\omega_{1,2}=0.5$ in both scenarios). The blue and red curves correspond to the unfiltered and filtered stages, respectively.

Figure 14 shows the resulting trajectories for the spiral reference trajectory in the case of two and three nodes, respectively. In both cases, a median filter with a sliding window of 20 samples is used to smooth the output of the fusion stage. In this example, the fusion of the three nodes yields a smaller tracking error trend when filtered.

The cascading technique can also be used for networks with a higher number of nodes. The adequate selection of the $\omega_{i}$ parameters of the different covariance intersection stages controls the relative importance of the nodes in the fusion process.

## 8. Conclusions

This paper presented a formulation for networks of IMUs, extending a representation of the multi-target problem discussed in the literature. The feasibility conditions are discussed based on the feasibility of the LMIs modeling the network. Moreover, the network with the consensus version (with the nodes exchanging information among them) is shown to have an edge over the non-consensus network (i.e., with independent nodes).

The results obtained show the advantage of the network consensus over the single IMU solutions. The limitations of the use of IMU networks are mainly related to the practical implementation and not to the theoretical framework. As referred in the introduction, the rigidity assumption in the relative positioning of the IMUs is likely not to hold permanently.

In this paper, it is implicitly assumed that data is acquired, synchronously, and immediately processed for reconstruction. However, it should be emphasized that data acquisition and reconstruction can occur sequentially in time, i.e., data is acquired during traveling, after which it can be downloaded and fed into the reconstruction stage. Moreover, this decoupled strategy allows the testing of different parameters for reconstruction. Also, synchronism in the data acquisition is likely not to hold for the whole duration of a mission (e.g., due to the mishandling of packages that can damage the devices, intrinsic failures, electronics interferences, etc.).

Future work involves multiple research directions, namely related to the following: (i) uncertainties, (ii) missing information, and (iii) data fusion strategies. A natural evolution is the testing of alternative formulations for the EKF, e.g., the discrete formulation (which, being computationally simpler, may lead to numerical problems). Also, more complex protocols need to be tested and stronger connections with the consensus theory need to be investigated. Essentially, this amounts to replacing the $\beta_{j}^{i}$ with adequate functions, being able to shape the interchange of information among the nodes.

The rigidity criteria that can be used to determine the performance bounds of the consensus approach will be a key research topic related to the presented framework. The non-rigid scenarios amount to unstructured uncertainties that need to be accommodated by the framework.

Also, the strategies to minimize any effects of missing data are to be investigated. This is mainly related to failures in the devices (or the network).

The advantage of combining covariance intersection and the consensus was illustrated in the simple example above when data is synchronized. Additional testing including asynchronous data scenarios, e.g., as in [30,31], is scheduled for future work.

## Data Availability

All software is available upon reasonable request to the author.

## Abbreviations

The following abbreviations are used in this manuscript:

IMU	Inertial Measurement Unit
LMI	Linear Matrix Inequalities
EKF	Extended Kalman Filter
UKF	Unscented Kalman Filter
CD-EKF	Continuous-Discrete Extended Kalman Filter
GPS	Global Positioning System
